# Can absence of cardiac activity on point-of-care echocardiography predict death in out-of-hospital cardiac arrest? A systematic review and meta-analysis

**DOI:** 10.1186/s13089-024-00360-x

**Published:** 2024-02-20

**Authors:** Omar Albaroudi, Bilal Albaroudi, Mahmoud Haddad, Manar E. Abdle-Rahman, Thirumoothy Samy Suresh Kumar, Robert David Jarman, Tim Harris

**Affiliations:** 1 Emergency Medicine, Hamad Medical Corporation, Doha, Qatar; 2 Emergency Medicine, Barts Health NHS Trust, London, UK; 3 Department of Public Health, College of Health Science, QU Health, Qatar University, Doha, Qatar; 4 Emergency Medicine, Royal Victoria Infirmary, Newcastle Upon Tyne, UK; 5 School of Health and Life Sciences, Teesside University, Middlesbrough, UK; 6 Queen Mary University of London, London, UK

**Keywords:** Emergency medicine, Resuscitation, Cardiac arrest, Ultrasound, Echocardiography, Point-of-care, Death, Survival, PoCUS, OHCA, CPR

## Abstract

**Aim:**

The purpose of this systematic review and meta-analysis was to evaluate the accuracy of the absence of cardiac motion on point-of-care echocardiography (PCE) in predicting termination of resuscitation (TOR), short-term death (STD), and long-term death (LTD), in adult patients with cardiac arrest of all etiologies in out-of-hospital and emergency department setting.

**Methods:**

A systematic review and meta-analysis was conducted based on PRISMA guidelines. A literature search in Medline, EMBASE, Cochrane, WHO registry, and ClinicalTrials.gov was performed from inspection to August 2022. Risk of bias was evaluated using QUADAS-2 tool. Meta-analysis was divided into medical cardiac arrest (MCA) and traumatic cardiac arrest (TCA). Sensitivity and specificity were calculated using bivariate random-effects, and heterogeneity was analyzed using *I*^2^ statistic.

**Results:**

A total of 27 studies (3657 patients) were included in systematic review. There was a substantial variation in methodologies across the studies, with notable difference in inclusion criteria, PCE timing, and cardiac activity definition. In MCA (15 studies, 2239 patients), the absence of cardiac activity on PCE had a sensitivity of 72% [95% CI 62–80%] and specificity of 80% [95% CI 58–92%] to predict LTD. Although the low numbers of studies in TCA preluded meta-analysis, all patients who lacked cardiac activity on PCE eventually died.

**Conclusions:**

The absence of cardiac motion on PCE for MCA predicts higher likelihood of death but does not have sufficient accuracy to be used as a stand-alone tool to terminate resuscitation. In TCA, the absence of cardiac activity is associated with 100% mortality rate, but low number of patients requires further studies to validate this finding. Future work would benefit from a standardized protocol for PCE timing and agreement on cardiac activity definition.

**Supplementary Information:**

The online version contains supplementary material available at 10.1186/s13089-024-00360-x.

## Introduction

Cardiac disease accounts for around 1 in 3 deaths in wealthier nations with 15% presenting initially in out-of-hospital cardiac arrest (OHCA). OHCA has a poor survival rate, averaging around 8%, with an average global incidence among adults of 55 OHCAs per 100,000 person-years [1, 2]. Survival is higher for shockable as compared to non-shockable rhythms, witnessed as opposed to unwitnessed arrests and for patients who receive bystander cardiopulmonary resuscitation (CPR) [3]. Improved rates of bystander CPR, the availability of automatic defibrillators, and advances in critical care have been associated with improved outcomes in some countries [4]. The outcome of patients with non-shockable rhythm remains low and depends on early recognition and correction of potentially reversible causes [5, 6]. Resuscitation from cardiac arrest demands significant resources and identifying patients with no chance of survival allows health care providers to focus their efforts appropriately [7]. Previous work has identified combinations of clinical parameters and end tidal carbon dioxide levels as predictive of futility [8, 9].

Point-of-Care Echocardiography (PCE) is increasingly used in the evaluation of patients in the Emergency Department (ED) in guiding the diagnosis and resuscitation of patients with acute breathlessness, shock, and cardiac arrest [10]. During the resuscitation of cardiac arrest, PCE and blood gas are key in identifying reversible causes of cardiac arrest. PCE can complement advanced life support (ALS) and its use has been integrated into the universal ALS algorithm [11]. PCE may also have a role in identifying patients for whom resuscitation is futile. Prior systematic reviews and meta-analyses addressed this issue and had methodologic differences in the selected population and outcomes. Four reviews combined the data on both traumatic and non-traumatic cardiac arrest in their meta-analysis [12–15]. Another review excluded the data on shockable rhythm [16]. All these previous reviews reported performance measures of PCE in predicting survival outcomes. This systematic review focused on the prediction of death, with subgroup analysis of medical versus traumatic cardiac arrest, irrespective of the cardiac rhythm.

The purpose of this systematic review and meta-analysis was to evaluate if the absence of cardiac motion on intra-arrest PCE predicts death. The endpoints were the sensitivity and specificity of the absence of cardiac motion for the absence of spontaneous return of circulation (ROSC), survival to hospital admission (SHA), and survival to hospital discharge (SHD) for adult patients with OHCA of all etiologies. The review question is described in Table 1.

**Table 1:** Review question

Population	Adult patients with out-of-hospital cardiac arrest (OHCA) or in-ED cardiac arrest (EDCA) irrespective of cause or rhythm
Intervention	Point-of-care echocardiography (PCE) during CA to identify cardiac standstill
Outcomes	▪ Termination of resuscitation (TOR): defined as no return of spontaneous circulation (ROSC) in the ED or upon ED arrival in prehospital studies ▪ Short-term death (STD): defined as no survival to hospital admission (SHA) or at 24 h ▪ Long-term death (LTD): defined as no survival to hospital discharge (SHD) or at 30 days ▪ Neurologically intact survival to hospital discharge (NISHD) Subgroups for analysis: ▪ Medical cardiac arrest (MCA) ▪ Traumatic cardiac arrest (TCA)

## Methods

This systematic review was designed in accordance with the Preferred Reporting Items for Systematic reviews and Meta-Analysis (PRISMA) statement [17] and was registered on the International Prospective Register of Systematic Reviews (PROSPERO, CRD42021179246).

### Data sources and searches

A comprehensive search of the literature was performed using Medline (PubMed), EMBASE (OvidSP), and Cochrane library in May 2021 and repeated in October 2023 from database inception to search date. A search for ongoing clinical trials was performed using ClinicalTrials.org and WHO registry. The search was conducted using MeSH (Medical Subject Headings) terms and search terms as shown in Additional file 1: Appendix S1. The initial search was supplemented by snowballing. The gray literature was searched using Google Scholar, OpenGrey, and the TRIP database. Emergency medicine and ultrasound journals were also hand searched. There were no limitations on the date of publication or the country of origin. The search was restricted to human studies and English language.

### Study selection

Two reviewers (TH, OA) independently conducted the search and identified studies for inclusion by reviewing the titles and abstracts. Consensus was then achieved by reading the full text of all the potentially eligible papers. Any discrepancy in study inclusion or exclusion was resolved by discussion between the reviewers, and independent search of a third reviewer (RDJ). Randomized controlled trials and observational cohort studies (prospective or retrospective) in prehospital or ED setting were included. Conference abstracts were only included if contained sufficient methods description for quality assessment, and sufficient data for analysis. Case reports, case series, reviews, guidelines, editorials, and letters were excluded. Studies involving in-hospital cardiac arrest (IHCA) or using transesophageal echocardiography (TEE) were excluded.

### Data extraction and quality assessment

A standardized data abstraction form was used to summarize studies (Table 2). Data were extracted independently by two authors (OA, TH) and verified by two reviewers (RDJ, TSK). Quality assessment of the included studies was performed using QUADAS-2 (Quality Assessment of Diagnostic Accuracy Studies) tool [18]. Attempts were made to contact authors to clarify methods and obtain missing data. The tool was applied by two reviewers (TH, OA) independently and any disagreement in quality scoring was resolved by independent assessment of a third reviewer (RDJ). QUADAS -2 tool allows customization of the signaling questions to assess papers included in this review. Our signaling questions are detailed in Additional file 1: Appendix S2 with the definitions of low and high risk of bias for each of the tool domains.

**Table 2:** Summary of the included studies

Author/year Country Design	Inclusion Exclusion Setting	Ultrasound timing Cardiac activity definition Operator	Machine Probe Windows	Outcomes
**Masoumi** **2021**	> 18 yo, OHCA & EDCA, non-traumatic cause, non-shockable rhythm	During the first 3 CPR pauses < 10 s, treating clinicians were not blinded to US findings except for cardiac motion	Philips Affiniti 70	*n =* 151 ROSC
Iran	Resuscitation < 4 min (*n =* 10), US not done (*n =* 9), other reasons (*n =* 5)	Any visible atrial, valvular, or ventricular movement, excluding movement of blood within cardiac chambers or isolated valve movement	Curvilinear	SHA
POS	2 urban EDs	5 EPs with > 6 years of experience in emergency echocardiography	SC	SHD
**Devia** **2020**	> 18 yo, OHCA and EDCA, non-traumatic cause, PEA rhythm	Not mentioned	SonoSite M-Turbo	*n =* 56 ROSC
Colombia	Trauma, referred to other institutions through administrative request	Not mentioned	Phased array	24-h survival
ROS	Single-center ED	Not mentioned	SC, PSLA, A4C or A5C	SHD
**Atkinson** **2019**	> 19 yo, OHCA, non-traumatic cause, non-shockable rhythm	Designated pauses (pulse/rhythm checks, intubation) minimized as per ACLS	Not mentioned	*n =* 180 ROSC
Canada	US not done (*n =* 43), < 19 yo, IHCA, DNR	Sustained coordinated contractility of LV, with visible valve movement	Curvilinear, Phased array	SHA
ROS	Tertiary center ED	Credentialed EPs (CPOCUS/CEUS/IFEM)	SC, PSLA, A4C	SHD
**Israr** **2019**	> 18 yo, OHCA, traumatic cause, PEA rhythm	Not mentioned	Not mentioned	*n =* 79
USA	US not done (*n =* 31), medical cause	Not mentioned	Not mentioned	SHA
ROS	2 level-1 trauma centers	Trauma surgeons	Not mentioned	SHD
**Lien** **2018**	OHCA, non-traumatic cause, shockable & non-shockable rhythm	Pulse/rhythm every 2-min check < 10 s	Toshiba SSA-550A	*n =* 177 ROSC
Taiwan	DNR (*n =* 56), US not done [29], US not complete [2], tracheostomy [8], neck tumors [2], neck operation, pregnancy	Not mentioned	Curvilinear	
POS	Tertiary center ED	10 EPs who attended basic emergency US training & 4 h focused training	SC	SHD
**Khunkhlai** **2017**	OHCA, non-traumatic cause, shockable & non-shockable rhythm	On ED arrivals and 5 repetitive scans every 2 min during CPR pause	Not mentioned	*n =* 63 ROSC
Thailand	Not mentioned	Cardiac wall AND/OR valvular movement	Not mentioned	SHA
POS	Single-center ED	Not mentioned	Not mentioned	30-day survival
**Chua** **2017**	> 21 yo, OHCA, non-traumatic cause, shockable & non-shockable rhythm	Pulse checks < 10 s. If the leader decided to continue resuscitation in < 10 s, US would cease	SonoSite Edge II & Terason	*n =* 101
Singapore	Pregnant, terminally ill, EDCA (*n =* 53), DNR [15], ROSC on ED arrival [18]	Not mentioned	Not mentioned	SHA
POS	Tertiary center ED	Senior residents and above completed training program (detailed in the paper)	Not mentioned	SHD
**Gaspari** **2016**	OHCA & EDCA, non-traumatic cause, non-shockable rhythm	Pulse/rhythm check at beginning of ACLS in ED and 2nd US at end of resuscitation	Not mentioned	*n =* 793 ROSC
USA Canada	Resuscitation ended after US (*n =* 106), no ACLS meds given (*n =* 42), DNR [8], incomplete timing data [3], unable to interpret US [1]	Any visible movement of the myocardium, excluding movement of blood within the cardiac chambers or isolated valve movement	Not mentioned	SHA
POS	Multicenter (20 EDs)	EPs credentialed in bedside US by their individual hospitals	SC, PSLA	SHD
**Kim** **2016**	Convenience sampling, > 18 yo, OHCA, non-traumatic cause, shockable & non-shockable rhythm	During pulse checks every 2 min < 10 s Patients were committed to 30 min of resuscitation	GE LOGIQ S6	*n =* 48 ROSC
Korea	No sonographer (*n =* 142), < 18 yo [7], trauma [24], drug intoxication [1]	Any detected atrial, valvular, or ventricular motion within the heart	Phased array	
POS	Tertiary center ED	2 senior residents & 3 EM specialists with ≥ 3 years of experience in emergency echo	SC, PS	SHD
**Zengin** **2016**	> 18 yo, OHCA & EDCA, non-traumatic cause, shockable and non-shockable rhythm	Femoral pulse check < 10 s, for 3 inspections	GE Logiq P6	*n =* 179 ROSC
Turkey	Trauma (*n =* 51), technical & anatomical reasons (*n =* 27), no sonographer (*n =* 7)	Any detected motion of the myocardium, ranging from visible VF to coordinated ventricular contractions	Tightly-curved	
POS	Single-center ED	2 senior doctors with 16 h of theoretical and applied focused echo training & 8 h of basic emergency US training	SC, PS, A4C	SHD
**Ozen** **2016**	Convenience sampling, > 18 yo, OHCA & EDCA, traumatic and non-traumatic cause, shockable and non-shockable rhythm	Pulse checks	Hitachi Aloka Prosound 6	*n =* 129 ROSC
Turkey	< 18 yo, pregnant, thoracic deformities or injuries 30 = rapid transfer to OR, missing personal data and high patient volume of the ED 18 = lost to follow-up	Not mentioned	Curvilinear	SHA
POS	Single-center ED	Senior EM residents with at least 2 years of clinical experience and EMAT US certification	SC	1-month survival
**Bolvardi** **2016**	> 18 yo, OHCA & EDCA, traumatic and non-traumatic cause, shockable and non-shockable rhythm	Not mentioned	Honda-Japan	*n =* 159 ROSC
Iran	Terminal illness, drowning, stroke, severe hypothermia	Any heart activity including the ventricles, galleries, valves, etc.	Curvilinear	
POS	Single-center ED	Physician who was not a member of resuscitation team and had no knowledge of initial rhythm	SC	
**Inaba** **2015**	OHCA & EDCA, traumatic cause and underwent resuscitative thoracostomy in ED	Just before or concurrent with thoracotomy	SonoSite S-FAST or M-Turbo	*n =* 180
USA	Emergent or urgent thoracotomy in OR, inadequate view (*n =* 7)	Organized, non-fibrillating contractions	Phased array	
POS	Single-center ED	PGY 2–4 EM residents under direct supervision, completed 16-h US course consisting of didactics and hands-on training and minimum 2 weeks of training in PoCUS	SC, PS	SHD
**Cebicci** **2014**	> 18 yo, OHCA & EDCA, traumatic and non-traumatic cause, shockable and non-shockable rhythm	Patient arrival	CHISON 8500	*n =* 410 ROSC
Turkey	No recorded rhythm or US at arrival (*n =* 73)	Not mentioned	Curvilinear	24-h survival
ROS	Single-center ED	4 EM specialist certified in emergency US and have enough experience	Not mentioned	
**Ferrada** **2014**	OHCA, traumatic cause and did not survive resuscitation	Pulse check < 10 s	Not mentioned	*n =* 14
USA	US not done (*n =* 23)	Not mentioned	Not mentioned	
ROS	Trauma center	Trained EPs, trauma attending surgeons, and residents in both specialties	SC, PSLA, PSSA, A4C	
**Cureton** **2012**	> 18 yo, OHCA, traumatic cause, non-shockable rhythm	Not mentioned	SonoSite MicroMaxx	*n =* 162
USA	US not done (*n =* 156)	Organized non-fibrillating motion	Curvilinear	SHA
ROS	University-based urban trauma center	Surgeon or EP with US training under direct supervision of FAST-credentialed EM attending or trauma surgeon	SC	SHD
**Aichinger** **2012**	Convenience sampling, > 18 yo, OHCA, non-traumatic cause, shockable and non-shockable rhythm	Rhythm/pulse check and after initial procedures (defibrillation, intubation, vascular access), multiple echoes allowed; CPR had to be continued for at least 15 min after initial echo	SonoSite 180 Plus (portable machine)	*n =* 42
Austria	< 18 yo, trauma	Any detected motion of the myocardium, ranging from visible VF to coordinated ventricular contractions	Micro-convex	ROSC on ED arrival
POS	2 emergency vehicles that are comparable to mobile ICUs on call 24 h per day	24 EPs with 2-h course in focused echo	SC	SHD
**Tomruk** **2012**	Convenience sampling, > 18 yo, OHCA and EDCA, traumatic and non-traumatic cause, shockable and non-shockable rhythm	Immediate during initial assessment	Chison 600 M	*n =* 149 ROSC
Turkey	Terminal illness, drowning, hanging, severe hypothermia	Any detected motion within heart, including atrial, valvular, and/or ventricular motion	Curvilinear	
POS	Single-center ED	EPs with theoretical and hands-on training on cardiac US	SC	
**Chardoli** **2012**	Convenience sampling, adult, OHCA, traumatic and non-traumatic cause, PEA rhythm	First pulse check < 10 s	Not mentioned	*n =* 100 ROSC
Iran	Not mentioned	Not mentioned; cardiac activity finding was not notified to leader to avoid bias in CPR duration	Not mentioned	
RCT	2 academic EDs	Emergency resident with course to achieve competence in echo for subxiphoid view in 10 s	SC	
**Hayhurst** **2011**	Convenience sampling, adult, OHCA, traumatic and non-traumatic cause, shockable and non-shockable rhythm	Rhythm check < 10 s	Not mentioned	*n =* 49 ROSC
UK	US done outside cardiac arrest period (*n =* 6), incomplete data	Not mentioned; US did not contribute to any decisions to stop ALS	Curvilinear, Phased array	SHA
POS	2 centers EDs	EPs or specialist trainees with Level 1 competency in emergency US. Extra sessions held for revision of 3 cardiac windows and assessed obtaining adequate picture in 10 s	SC, PSLA, A4C	SHD
**Tarmey** **2011**	> 18 yo, OHCA & EDCA, traumatic cause, shockable and non-shockable rhythm	Not mentioned	Not mentioned	*n =* 24 ROSC
Afghanistan	Declared dead prior to arrival, or arrested only after withdrawal of active management, US not done (*n =* 28)	Not mentioned	Not mentioned	
POS	Military trauma center	Not mentioned	Not mentioned	SHD
**Breitkreutz** **2010**	OHCA, non-traumatic cause, non-shockable rhythm	Pulse check < 10 s	Hand-held US (modified Tringa by Esaote) + 3.5–5 MHz probe	*n =* 88
Germany	Not mentioned	Not mentioned	Standard US (SonoSite i-Look 15) + curved array probe	ROSC on ED arrival
POS	4 EMS (Emergency Medical Services)	EPs trained in peri-resuscitation echo (FEEL program). EP was specialist in cardiology, IM, surgery, anesthesia, or pediatrics with an additional sub-specialization in prehospital EM	SC, PS, A4C	
**Schuster** **2009**	OHCA and EDCA, traumatic cause, PEA rhythm	Not mentioned	Philips EnVisor	*n =* 27 ROSC
USA	Not mentioned	Organized non-fibrillating contractile activity with decrease in chamber size	Curvilinear, Phased array	
ROS	Level-1 trauma center	Trained senior surgeon or EM resident under direct supervision of FAST-credentialed trauma surgeon or EP	SC, PS	SHD
**Salen** **2005**	Convenience sampling, > 16 yo, OHCA and EDCA, non-traumatic cause, non-shockable rhythm	Initial US on presentation and sequential examinations every 3–5 min during carotid pulse checks	Not mentioned	*n =* 70 ROSC
USA	Not mentioned	Any detected motion within the heart: atrial, valvular, or ventricular	Curvilinear, Phased array	SHA
POS	4 academic EDs	EPs	SC, PSLA	SHD
**Tayal** **2003**	OHCA, non-traumatic cause, PEA rhythm	Not mentioned	Shimadzu SDU-400	*n =* 20 ROSC
USA	Not mentioned	Ventricular wall motion	Not mentioned	
POS	Tertiary center ED	Trained EPs with 20-h emergency ultrasound course, followed by continuous quality improvement reviews and direct feedback	SC, PS, A4C	SHD
**Salen** **2001**	Convenience sampling, > 18 yo, OHCA, non-traumatic cause, shockable and non-shockable rhythm	Pulse check < 10 s repeated with any change in rhythm	Pie Medical Scanner 200 and GE RT3200 Advantage II	*n =* 102
USA	Not mentioned	Not mentioned	Curvilinear	SHA
POS	2 community hospital EDs	EPs, residents and attendings with 4-h trauma US course during which focused cardiac US was taught and practiced on simulator models	SC, A4C	
**Blaivas** **2001**	Convenience sampling, > 18 yo, OHCA, non-traumatic cause, shockable and non-shockable rhythm	On patient arrival and during pulse check < 10 s	Aloka 2000	*n =* 169
USA	< 18 yo, trauma, non-cardiac cause (drug overdose)	Not mentioned	Curvilinear, Phased array	SHA
POS	Urban community hospital ED	US trained and credentialed EPs (resident and attendings)	SC, PS	

POS: Prospective Observational Study, ROS: Retrospective Observational Study. OHCA: Out-of-Hospital Cardiac Arrest, EDCA: in-ED Cardiac Arrest. SHA: Survival to Hospital Admission, SHD: Survival to Hospital Discharge. SC: SubCostal, PSLA: ParaSternal Long Axis, A4C: Apical 4 Champers, yo: years old, EP: Emergency Physician, DNR: Do Not Resuscitate

### Data synthesis and analysis

For analysis, a true positive was defined as a patient with the outcome of interest (TOR, STD, or LTD) and cardiac standstill on PCE. Hence, the condition being tested was death, and a positive test was cardiac standstill on PCE. The reported sensitivity (true-positive rate) was the proportion of patients who died and in whom the PCE identified cardiac standstill. The reported specificity (true-negative rate) was the proportion of patients who survived and accurately identified by PCE as having cardiac activity. All studies that provided data to enable the calculation of performance estimates of PCE to predict death were used in the meta-analysis. Point estimates for each study and pooled estimates with 95% confidence intervals of sensitivity and specificity were calculated using bivariate random-effects modeling. Forest plots were used to display the results. Heterogeneity across studies was analyzed using the Higgins’ I^2^ statistic which ranges between 0 and 100%; I^2^ of 75% or higher indicated high heterogeneity [19]. Deeks funnel plot was used to identify evidence of publication bias in studies of diagnostic performance. The meta-analysis was performed on Stata 16 (StataCorp, 2019) using the user-defined program Midas [20].

## Results

### Search results

**Fig. 1:**
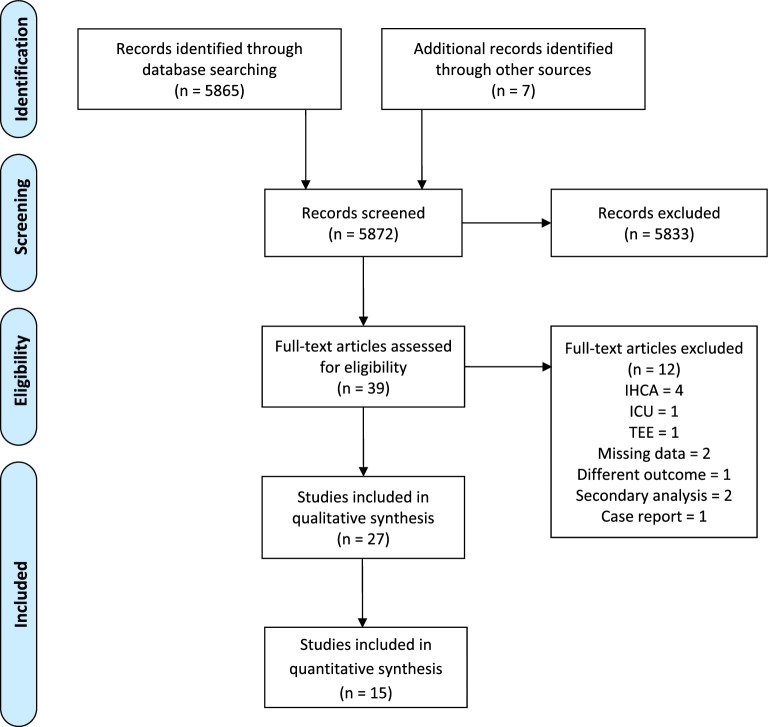
PRISMA flow diagram

Literature search results are displayed in Fig. 1, with 5872 studies screened, 39 full-text papers reviewed, and 27 included in the qualitative synthesis of which sufficient data were identified in 15 for meta-analysis. Further full-text screening excluded 12 papers that did not meet the inclusion criteria. Two excluded papers were secondary analysis of other included studies [21, 22]. Four excluded studies focused on in-hospital cardiac arrest [23–26], and one was conducted in an intensive care unit setting [27]. One study used TEE to identify intracardiac thrombus [28], and another one looked into the impact of prehospital echocardiography on treatment decisions [29]. We also excluded two abstracts with insufficient data for analysis [30, 31], and a case report [32].

### Study characteristics

A total of 27 studies (3657 patients) were included in this systematic review [33–59]. Two studies were performed in a prehospital setting [51, 54], and the remaining involved PCE performed in the ED. We contacted the authors for 11 studies to inquire about the study population, clarify the ultrasound protocol, and obtain missing outcome data [36, 38, 39, 41–44, 47, 50, 51, 54]. All included studies were published between 2001 and 2021. The study design for all but one study was observational cohort, 19 of which were prospective [33, 37–45, 48, 51–54, 56–59] and seven were retrospective studies [34–36, 46, 47, 49, 55]. There was one randomized controlled trial [21]. Seven studies conducted in at least two centers [33, 35, 43, 50, 53, 56, 58], with the largest recruiting from 20 centers [43]. All 27 studies included OHCA patients. Twelve studies also included EDCA patients [33, 34, 40, 41, 43–45, 47, 48, 52, 55, 56]. Six studies included only patients in TCA [35, 45, 46, 49, 52, 55], and 15 studies only MCA patients. The remaining six studies included all cardiac arrest patients regardless of the cause [41, 44, 47, 48, 50, 53]. While 14 studies included both shockable and non-shockable rhythms, 11 studies included patients where the initial presenting rhythm was non-shockable [33–36, 43, 49, 50, 54–57], five of which included only patients with pulseless electrical activity [34, 35, 50, 55, 57]. All participants in the studies were adults aged over 16 years. All studies used at least the subcostal window, except five studies which failed to describe which PCE windows were used [35, 38, 39, 47, 52]. The most frequently used ultrasound probe was curvilinear [33, 36, 37, 41, 44, 47–49, 53–56, 58, 59], then phased array probe [34, 36, 42, 45, 53, 55, 56, 59], and eight studies did not specify which probes were used [35, 38, 39, 43, 46, 50, 52, 57].

### Quality assessment

The results of the QUADAS2 assessment are presented in Table 3. There was considerable variation in study methods. Eighteen studies were rated as high risk of bias for patient selection, mainly because of convenience sampling and exclusion criteria (e.g., due to anatomical or technical difficulties). The PCE protocols varied between studies, which is reflected in scoring the index test. Thirteen studies failed to a priori define how the presence or absence of cardiac activity was assessed [34, 35, 37, 39, 41, 46, 47, 50, 52–54, 58, 59]. Two studies were rated high risk of bias due to loss of patient data [40, 41].

**Table 3:**
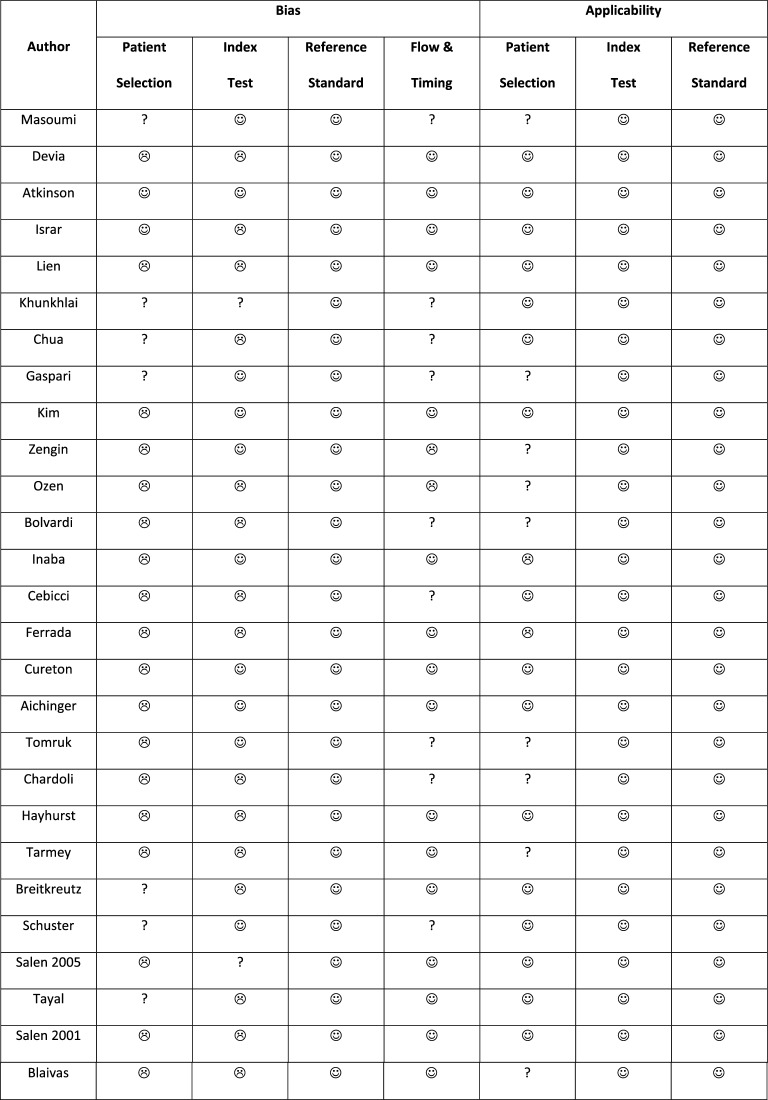
Quality assessment of the included studies (QUADAS-2)

### Systematic review

Each study reported one or more of the following outcomes: ROSC (20 studies), SHA (12 studies), 24-h survival (two studies), 30-day survival (two studies), and SHD (17 studies). The results of these outcomes are tabulated in Additional file 1: Appendix S3. Three studies reported neurologically intact SHD for three patients, all of which had cardiac motion on PCE during MCA [42, 51, 56]. PCE assessment was done during pulse and rhythm check in all studies that specified the timing. There was a variable number of PCE assessments during resuscitation period. While most studies reported their outcomes based on single PCE assessment, five studies reported increased odds of poor outcome if persistent absence of cardiac activity was noted on several assessments [33, 37, 38, 42, 51]. Masoumi et al. (*n =* 151) reported 91% specificity for TOR in patients with cardiac standstill on three ultrasound assessments during the first three CPR pauses, compared to 61% specificity for TOR if no cardiac activity on the first assessment [33]. Kim et al. (*n =* 48) evaluated the correlation between serial echocardiographic assessments and ROSC and found 25% specificity for TOR in patients with cardiac standstill on the initial sonographic assessment, which increased to 85%, 96%, and 100% at 6, 8, and 10 min of serial cardiac standstill. [42]. Definition for cardiac activity varied between the studies and ranged from any detected motion to organized wall motion. Khunkhlai et al. (*n =* 63) showed a slight increase in sensitivity and decrease in specificity of TOR and STD if both wall and valvular motion were absent (TOR sensitivity 100%, specificity 65%; STD sensitivity 74%, specificity 70%), compared to the absence of only wall or valvular motion (TOR sensitivity 96%, specificity 76%; STD sensitivity 67%, specificity 80%) [38].

### Meta-analysis

Meta-analysis for the included studies was subclassified into MCA and TCA groups. Studies that included both medical and traumatic cardiac arrest with no available data for each were excluded from this analysis. The small number of studies in the TCA group with low numbers of reported events did not allow for a meta-analysis. As a result, 15 studies (2239 patients) were included in this meta-analysis for the MCA group.

**Fig. 2:**
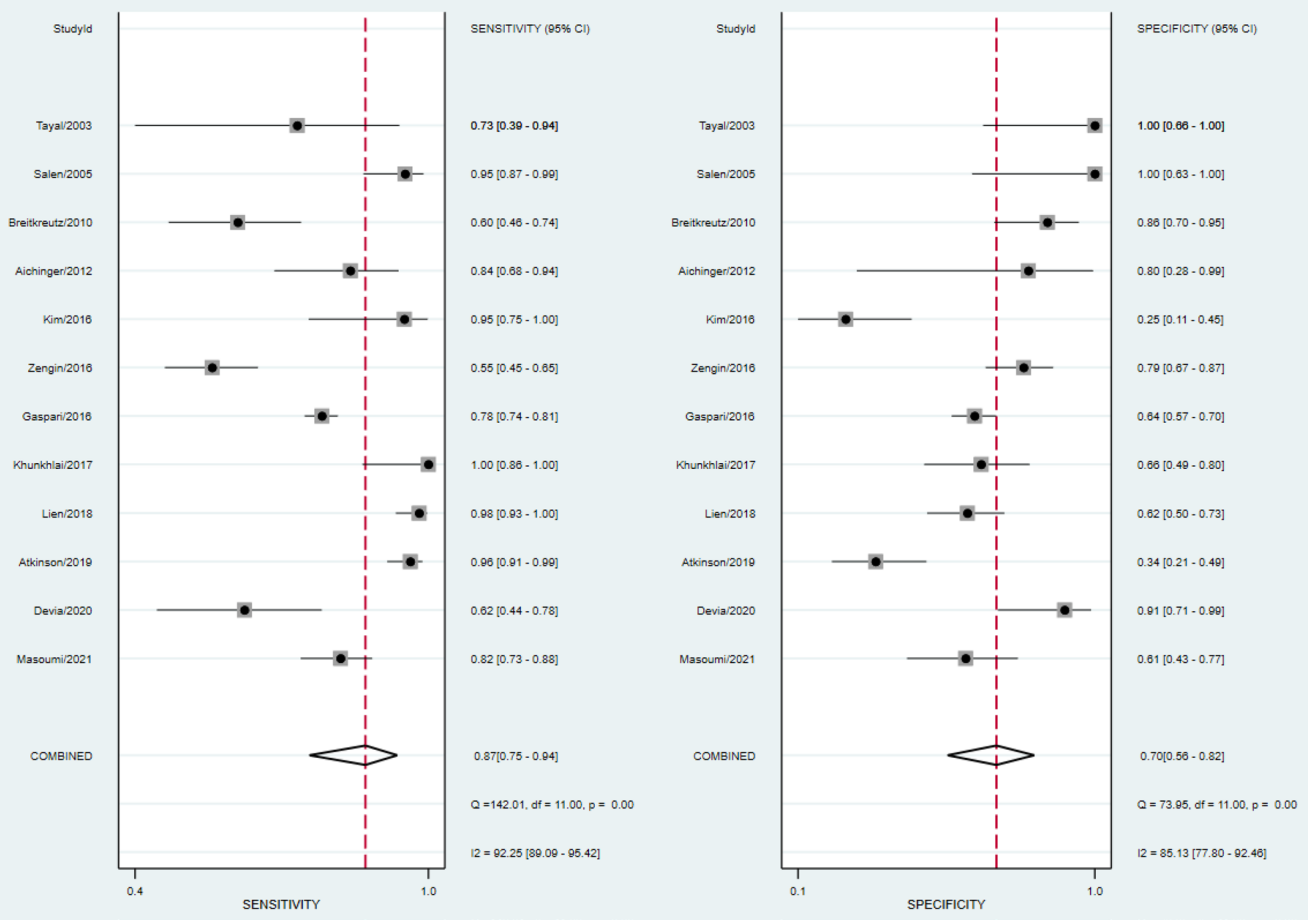
Forest plot of sensitivity and specificity for TOR outcome in MCA group

**Fig. 3:**
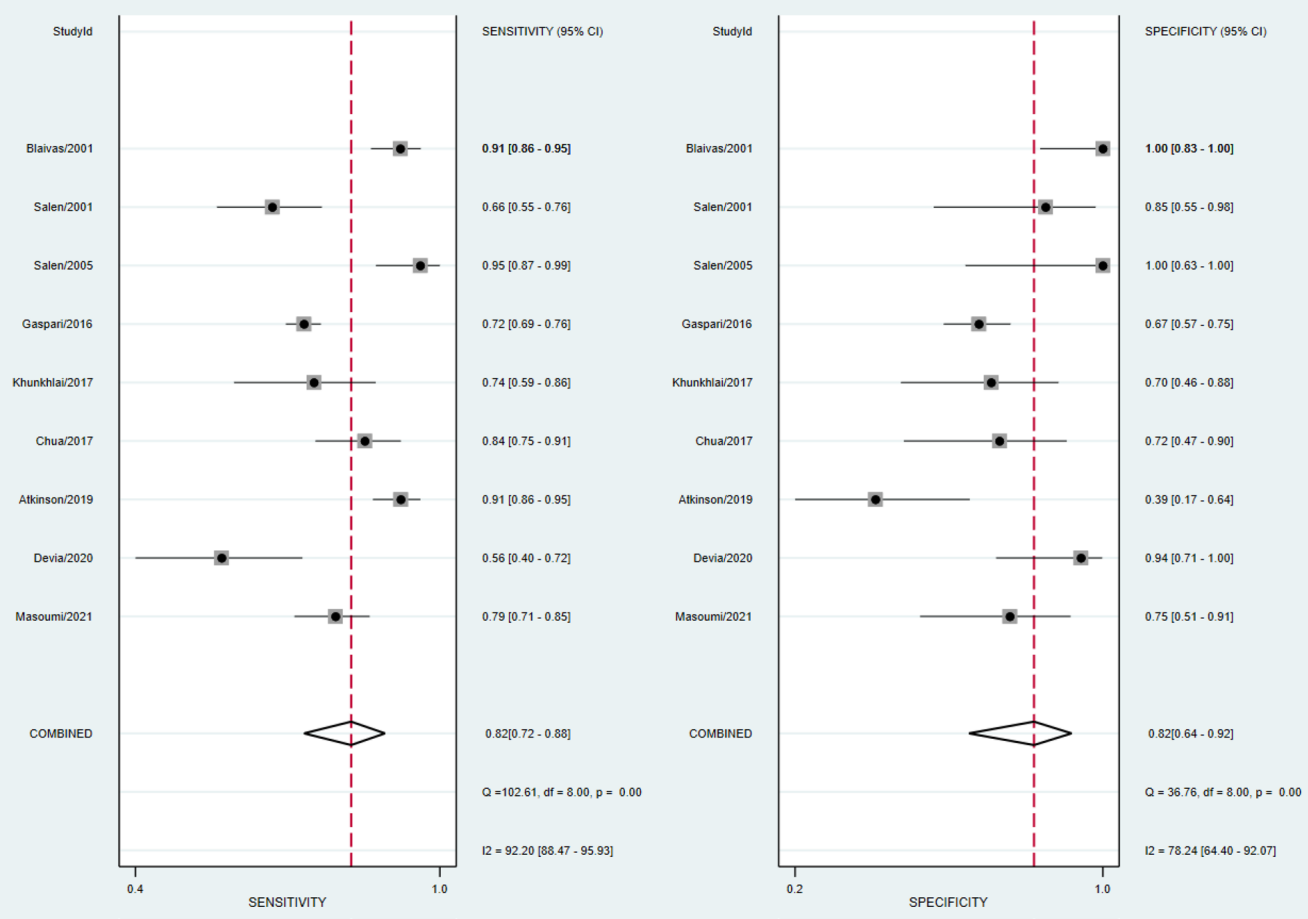
Forest plot of sensitivity and specificity for STD outcome in MCA group

**Fig. 4:**
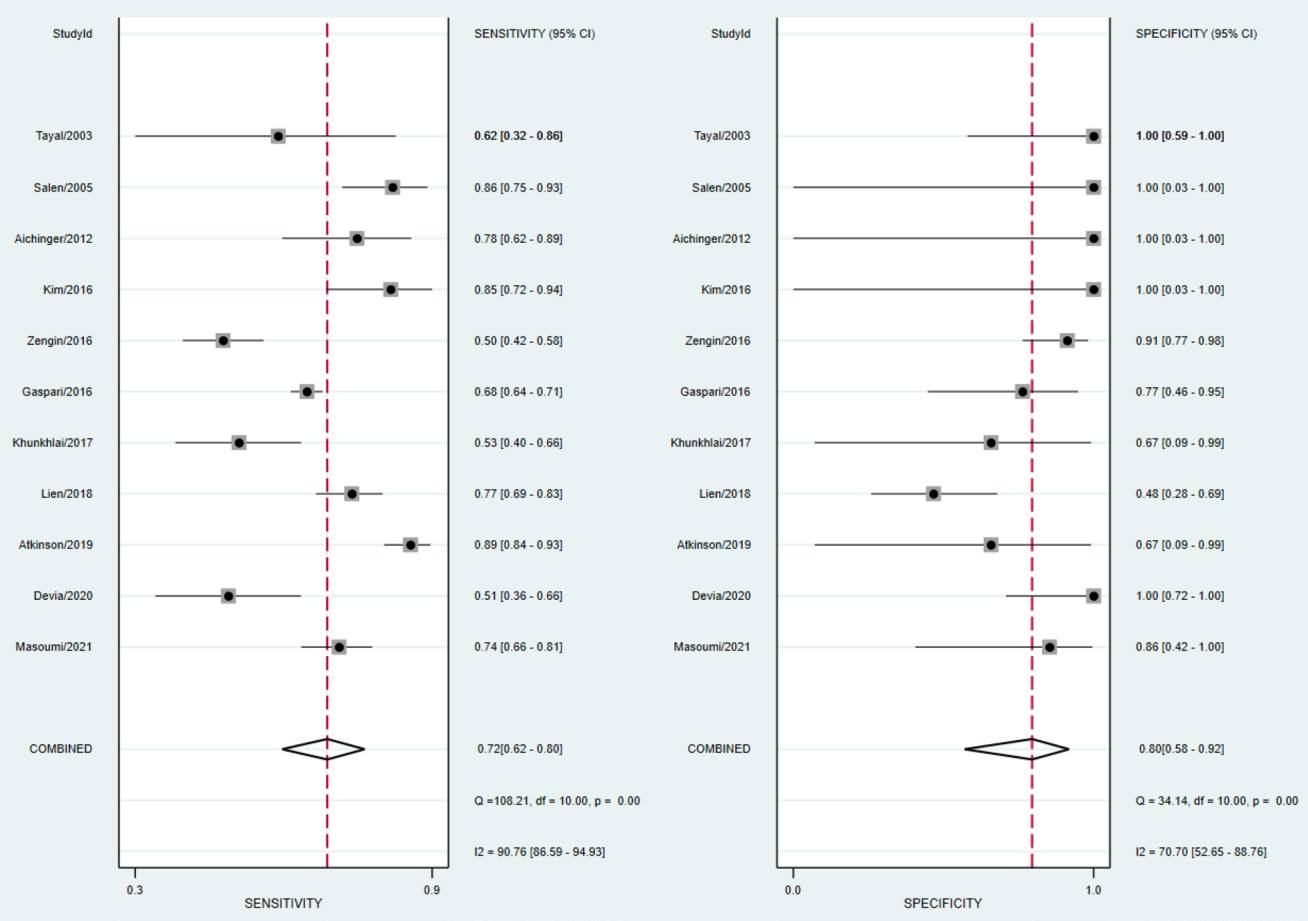
Forest plot of sensitivity and specificity for LTD outcome in MCA group

The absence of cardiac activity on PCE in MCA group had a pooled sensitivity of 87% [95% CI 75–94%] and specificity of 70% [95% CI 56–82%] to predict TOR. Pooled sensitivity to predict STD was 82% [95% CI 72–88%] and specificity 82% [95% CI 64–92%]. To predict LTD, pooled sensitivity was 72% [95% CI 62–80%] and specificity 80% [95% CI 58–92%]. There was a substantial heterogeneity of the results, with I^2^ exceeding 75% for both sensitivity and specificity analysis. The forest plots of the previous results are shown in Figs. 2, 3, 4. Positive and negative likelihood ratios are reported in Additional file 1: Appendix S4. There was no evidence of publication bias as demonstrated by Deeks’ funnel plot in Additional file 1: Appendix S5.

Although meta-analysis was not feasible for TCA group, the rate of LTD was 100% (358/358) for patients without cardiac activity, and 90% (103/114) for patients with cardiac activity on PCE. Thus, no patient survived to hospital discharge if there was an absence of cardiac activity on PCE during traumatic cardiac arrest.

## Discussion

The findings of this systematic review suggest that the predictive value of cardiac standstill on PCE for death differs between medical and traumatic cardiac arrest. In MCA, the specificity for long-term death (LTD) was 80% (58–92%); thus, a significant number of false-positive cases (22/1779 reported cases) were identified where patients with absent cardiac activity survived to hospital discharge. Therefore, PCE cannot be used as a sole tool to predict death and direct the cessation of resuscitation in MCA. The specificity for LTD in TCA was 100%; thus, all patients without cardiac activity (358 patients) failed to survive to hospital discharge, and consequently died. However, the low numbers of patients preclude any firm conclusions.

The sensitivity and specificity to predict TOR in MCA were 87% [95% CI 75–94%] and 70% [95% CI 56–82%], respectively. However, it is worthy to note that Atkinson et al. (*n =* 180) reported a higher sensitivity and lower specificity of 96% and 34%, respectively [36]. The definition of cardiac activity (sustained coordinated contractility of left ventricle, with visible valve movement) in this study may explain these findings. Another discrepancy was also remarkable in Zengin et al. (*n =* 179) which reported the lowest sensitivity to predict TOR and LTD [40]. The high proportion of false-negative cases, patients who died despite identified cardiac activity on PCE, may reflect a wider definition of cardiac activity (any detected motion of the myocardium) or a less-experienced clinician sonographer group.

In contrast to previous meta-analyses, this study reported the utility of PCE findings during cardiac arrest as predictor of death (TOR, STD, and LTD), as opposed to survival (ROSC, SHA, and SHD), reflecting the question asked by clinicians when observing no cardiac motion. The meta-analysis reported on MCA and included all rhythms. The latter approach was taken as rhythm changes frequently during cardiac arrest, as does the timing of PCE in the analyzed papers, so the clinician sonographers could have timed their PCE to coincide with a certain rhythm in studies where the timing of the PCE was not protocolized.

Since previous meta-analyses used reversed outcome and test definitions, their sensitivity can be compared to our specificity. A recent systematic review evaluated PCE in predicting survival in non-traumatic non-shockable OHCA and reported pooled sensitivity of 60% for ROSC and 74% for SHD [16]. The exclusion of shockable rhythm may explain the lower sensitivity, as compared to the 70% specificity of TOR and 80% for LTD in this analysis. Two previous systematic reviews analyzed the data for both MCA and TCA with no subgroup analysis for each group provided and reported a higher sensitivity of 91% and 95% for ROSC which can be explained by the inclusion of traumatic arrest [14, 15]. The systematic review reported here had a greater heterogeneity in quality assessment compared to previous systematic reviews, which may reflect a more rigorous application of the QUADAS tool to identify any risk of bias. A more extensive literature review to include all the eligible studies was also notable.

A recent systematic review evaluating PCE as a predictor of death in TCA showed findings consistent with this analysis [60]. A previous systematic review investigated the prognostic association of different factors with survival and found that the most important predictors of SHD were the presence of cardiac motion on ultrasound (odds ratio 33.9, 95% CI 1.8–613.4) and shockable initial rhythm (odds ratio 7.2, 95% CI 5–10.4) [61]. In TCA, cardiac activity on PCE may be regarded as an extreme of shock. After ruling out obstructive causes of shock (cardiac tamponade and tension pneumothorax), the absence of any cardiac activity may imply unsalvageable condition as the myocardium has been exposed to a profound hypoxic insult to the point of no coordinated cellular activity. However, evidence of organized activity might indicate profound shock where aggressive resuscitation can potentially recover cardiac output and subsequently achieve survival.

Despite the proposed benefit of PCE in assisting clinicians in defining the etiology and predicting outcome of OHCA, the potential harm of intra-arrest PCE warrants consideration. Two small prospective observational studies identified that PCE use is associated with longer duration of pulse checks [62, 63]. However, another study suggested that the implementation of a structured ultrasound protocol reduced the duration of CPR interruptions [64]. The study protocol consisted of three sequential scans that evaluated for reversible causes in the first two CPR pauses, and cardiac activity in the 3rd pause. Other authors reported that pre-pause imaging (placing the transducer during CPR to identify the cardiac window) was associated with significant decrease in CPR pause time [65].

### Limitations

There are several limitations to this systematic review. The literature search was limited to English language. The majority of the included studies were observational cohort studies, which have inherent potential for bias and confounding. The lack of consecutive sampling puts the studies in the risk of selection bias, with many studies depending on the availability of a sonographer to recruit patients. The lack of blinding of cardiac activity on PCE has the potential to bias the clinical outcome, and overestimate the prognostic value of PCE, by increasing the association of cardiac standstill on PCE and death. Two studies found that patients with cardiac motion received longer length of resuscitation than those without (Atkinson: 27 min vs. 12 min, Gaspari: 18 min vs. 12 min) [36, 43]. Another two studies involved effort to overcome this confounding by continuing resuscitation at least 30 min [42] or at least 15 min after initial PCE [51]. This allowed these studies to assess the association between subsequent scan findings and death. The first study (*n =* 48) reported that in 18 patients with subsequent cardiac standstill ≥ 10 min, no one had ROSC. The second study was performed in the prehospital environment (*n =* 42) and reported higher ROSC rate of 57% (4/7) when cardiac activity presented in all performed echocardiographic assessments during resuscitation versus 40% (4/10) if cardiac activity detected in only the first echocardiography.

There was a considerable heterogeneity in the methodology between the included studies with different inclusion/exclusion criteria. The largest multicenter study (Gaspari et al.) included non-traumatic non-shockable OHCA and EDCA but did not include patients with brief resuscitation efforts of less than 5 min [43], which may had an effect on the overall low survival rate in this study (ROSC 26%, SHD 1.6%). Different ultrasound scanning protocols were also reported, with variety of ultrasound machines, transducers, and windows to evaluate for cardiac activity. Hayhurst et al. (*n =* 49) reported that the most successful window in obtaining adequate view within 10 s was the subxiphoid window (95%, 38/40), followed by parasternal (85%, 17/19) and apical window (50%, 2/4) [53].

Different timing for PCE assessments and variety of definitions for cardiac activity were used within the studies, which reflect the lack of standardized criteria in the literature. A secondary analysis of the study by Gaspari suggested that organized activity (contractions with changes in ventricular dimensions) is associated with higher survival rate (ROSC 65%, 49/75) compared to disorganized activity (agonal twitching) (ROSC 39%, 37/95) [21]. Additionally, the accuracy of ultrasound is known to be operator dependent, and each study required a differing level of training and clinical experiences. The inter-rater reliability for ultrasound interpretation was not reported in most studies; however, Gaspari et al. reported a substantial agreement (Cohen’s kappa = 0.63) [43]. Another survey study reported only moderate agreement of cardiac standstill (Krippendorff's alpha = 0.47) among 127 emergency medicine, critical care, and cardiology physicians shown 15 sonographic video clips [66]. Valvular flutter from mechanical ventilation and profound bradycardia had the most interobserver disagreement. This demonstrates the influence of inconsistent definition of cardiac standstill on the results, especially if interpreted with unskilled sonographer.

This methodological heterogeneity and risk of bias precluded ILCOR (International Liaison Committee on Resuscitation) from conducting meta-analysis in their systematic review on MCA, which included both out-of-hospital and in-hospital settings with no restriction on cardiac rhythm [67]. The main culprits were the wide variability in the definition of cardiac motion, the in timing of PCE assessment, and the confounding from “self-fulfilling prophecy,” when clinicians involved with the TOR decision were not blinded to the PCE findings. The authors concluded that the evidence for PCE as prognostic tool is of very low certainty.

## Conclusion

The absence of cardiac activity on intra-arrest PCE for MCA predicts a poor prognosis but is not a stand-alone tool to predict death and thus guide the cessation or continuation of a resuscitation. In TCA, the absence of cardiac activity is associated with a 100% mortality rate, but low numbers of included subjects indicate that further research is required before PCE findings are used as a stand-alone tool upon which to guide cessation of resuscitation. The methodological and reporting heterogeneity between studies hampers firm conclusions. Future work would benefit from a standardized protocol for intra-arrest PCE timing and definition of absent cardiac activity, and should focus on longer-term outcomes, such as 30–90-day survival with no or minimal disability.

### Supplementary Information

**Additional file 1.** Appendix S1, S2, S3, S4, S5.

## Data Availability

Data are presented in the main paper and the Additional file 1: Appendix.
