# Supplementary material for: Can absence of cardiac activity on point-of-care echocardiography predict death in out-of-hospital cardiac arrest? A systematic review and meta-analysis
Source: Ultrasound J. 2024 Feb 20;16:10. doi: 10.1186/s13089-024-00360-x (PMC10879065; doi:10.1186/s13089-024-00360-x)

*Appendix 1.*

| MeSH terms | ("Point-of-Care Systems"[Mesh] OR "Point-of-Care Testing"[Mesh]) AND ("Echocardiography"[Mesh] OR "Ultrasonography"[Mesh]) AND ("Heart Arrest"[Mesh] OR "Advanced Cardiac Life Support"[Mesh]). n = 92 |
| --- | --- |
| Search terms | (ultraso* OR sonograph* OR echocardiograph* OR POCUS) AND (arrest OR “life support” OR ACLS OR resuscitation OR CPR) AND (predict* OR survival OR outcome). n = 5773 |

*Appendix 2.1. Signaling questions for the assessment of risk of bias*

Patient Selection

1. Was a consecutive or random sample of patients enrolled?
2. Was a case-control design avoided?
3. Did the study avoid inappropriate exclusions?
4. Are there clear patient selection criteria that are rigorously applied?
5. Do the patients selected for study reflect patients who will receive PCE in practice?

Index Test

1. Were the PCE results interpreted & recorded without knowledge of outcome of cardiac arrest?
2. If a threshold was used, was it pre-specified? Was there a priori specified agreement which windows to use & how to assess & report cardiac function?
3. Did all patients receive PCE?
4. Were the methods of performing PCE adequately described?
5. Are the numbers accurately presented and were the results presented clearly with correct statistical tests?
6. Was the PCE performed by practitioners having similar clinical information to ‘real life’ practice?
7. Were equivocal or technically impossible scans reported (and how)?

Reference Standard

N/A

Flow and Timing

Were all patients accounted for and included in the analysis?

*Appendix 2.2. Definition of low and high risk of bias:*

Patient selection: Low risk of bias was defined as the patients being selected in consecutive sampling. Studies that used convenience sampling were considered to have a high risk of bias. Any inappropriate exclusion was also considered high risk for bias.

Index test: The index test was defined as the PCE performed during cardiac arrest. We considered the index test as low risk of bias if performed with a pre-defined technique (windows, probes, time of ultrasound, and level of operator), and a predefined cardiac activity. Studies that did not include detailed description of the ultrasound technique or did not define cardiac activity were considered as high risk of bias. Most studies did not report the duration of ultrasound, so we did not count it in the scoring of the index test. Though we recognize this as a potential risk of bias.

Reference standard: We defined the reference standard as the clinical outcome, i.e., either ROSC or death. Since death is intuitively the same, we do not have any signaling question that can be applied. That makes all studies considered to have low risk of bias.

Flow and timing: Here we have only one applicable signaling question in the scoring of the flow and timing. Studies that accounted for and included all patients in the analysis were considered to have low risk of bias. Any inappropriate exclusion from analysis was considered as high risk of bias.

*Appendix 3. Outcome results of the included studies*

*Abbreviations:* ***ROSC****: Return Of Spontaneous Circulation,* ***SHA****: Survival to Hospital Admission,* ***SHD****: Survival to Hsopital Discharge.* ***PCA****: Positive Cardiac Activity,* ***NCA****: Negative Cardiac Activity.*

| **Author Year** | ROSC | SHA  24-hr survival | SHD  1-month survival |
| --- | --- | --- | --- |
| **Masoumi 2021**  n=151 | ROSC  PCA first echo = 22/43 NCA first echo = 14/108  PCA any echo = 33/57  NCA all echos = 3/94  PCA all echos = 10/11 NCA any echo = 26/140 | SHA  PCA first echo = 15/43 NCA first echo = 5/108 | SHD  PCA first echo = 6/43 NCA first echo = 1/108 |
| **Devia 2020**  n=56 | ROSC  PCA = 20/33 NCA = 2/23 | 24-hr survival PCA = 16/33 NCA = 1/23 | SHD  PCA = 11/33 NCA = 0/23 |
| **Atkinson 2019**  n=180 | ROSC PCA = 16/21 NCA = 31/159 | SHA PCA = 7/21 NCA = 11/159 | SHD PCA = 2/21 NCA = 1/159 |
| **Israr 2019**  n=79 |  | SHA PCA = 5/22 NCA = 2/57 | SHD PCA = 0/22 NCA = 0/57 |
| **Lien 2018**  n=177 | ROSC PCA = 45/47 NCA = 28/130  PCA <4min = 0/1  NCA <4min = 6/13  PCA 4-6min = 1/2  NCA 4-6min = 13/20  PCA 6-8min = 5/5  NCA 6-8min = 8/23  PCA 8-10min = 12/12  NCA 8-10min = 1/24  PCA 10-12min = 16/16  NCA 10-12min = 0/32  PCA 12-14min = 10/10  NCA 12-14min = 0/16  PCA 14-16min = 1/1  NCA 14-16min = 0/2 |  | SHD PCA = 12/47 NCA = 13/130 |
| **Khunkhlai 2017**  n=63 | ROSC PCA (wall OR valve) = 29/30 NCA (wall OR valve) = 9/33 PCA (wall AND valve) = 25/25 NCA (wall AND valve) = 13/38 NCA all echos = 0/24 | SHA PCA (wall OR valve) = 16/30 NCA (wall OR valve) = 4/33 PCA (wall AND valve) = 14/25 NCA (wall AND valve) = 6/38 | 30-days survival PCA (wall OR valve) = 2/30 NCA (wall OR valve) = 1/33 |
| **Chua 2017**  n=101 |  | SHA PCA = 13/26 NCA = 5/75 |  |
| **Gaspari 2016**  n=793 | ROSC PCA = 134/263 NCA = 76/530 | SHA PCA = 76/263 NCA = 38/530 | SHD PCA = 10/263 NCA = 3/530 |
| **Kim 2016**  n=48 | ROSC PCA first echo = 7/8 NCA first echo = 21/40  PCA any echo @6min = 24/24 NCA all echos @6min = 4/24  PCA any echo @8min = 27/29 NCA all echos @8min = 1/19  PCA any echo @10min = 28/30 NCA all echos @10min = 0/18  PCA any echo @12 min = 28/32 NCA all echos @12min = 0/16 |  | SHD PCA = 1/8 (CPC 1) NCA = 0/40 |
| **Zengin 2016**  n=179 | ROSC PCA: 55/104 NCA: 15/75 |  | SHD PCA: 32/104 NCA: 3/75 |
| **Ozen 2016**  n=129 | ROSC PCA = 56/77 NCA = 3/52 | SHA PCA = 43/77 NCA = 1/52 | 1-month survival PCA = 8/77 NCA = 0/52 |
| **Bolvardi 2016**  n=159 | ROSC  PCA = 41/49 NCA = 15/110 |  |  |
| **Inaba 2015**  n=180 |  |  | SHD PCA = 9/54 NCA = 0/126 |
| **Cebicci 2014**  n=410 | ROSC PCA = 74/81 NCA = 5/329 | 24-hour survival PCA = 74/81 NCA = 5/329 |  |
| **Ferrada**  **2014**  n=14 | ROSC  PCA = 0/0  NCA = 0/14 |  |  |
| **Cureton 2012**  n=162 |  | SHA PCA = 6/20 NCA = 1/142 | SHD PCA = 0/20 NCA = 0/142 |
| **Aichinger**  **2012**  n=42 | ROSC on ED arrival PCA first echo = 4/10 NCA first echo = 1/32 PCA all echos = 4/7 NCA any echo = 1/35 |  | SHD PCA = 1/10 (full neurologic recovery) NCA = 0/32 |
| **Tomruk 2012**  n=149 | ROSC PCA = 19/27 NCA = 55/122 |  |  |
| **Chardoli 2012**  n=100  Group A (US) = 50 Group B (ACLS) = 50 | ROSC: Group A = 17/50 PCA = 17/39 NCA = 0/11 Group B = 14/50 |  |  |
| **Hayhurst 2011** n=49 | ROSC PCA = 11/20 NCA = 1/29 | SHA PCA = 4/20 NCA = 1/29 | SHD *PCA: not mentioned* NCA: 0/29 |
| **Tarmey 2011**  n=24 | ROSC PCA = 6/6 NCA = 0/18 |  | SHD PCA = 2/6 NCA = 0/18 |
| **Breitkreutz 2010**  n=88 | ROSC on ED arrival PCA = 30/51 NCA = 5/37 |  |  |
| **Schuster 2009**  n=27 | ROSC PCA = 3/12 NCA = 0/15 |  | SHD PCA = 0/12 NCA = 0/15 |
| **Salen 2005**  n=70 | ROSC PCA = 8/11  NCA = 0/59 | SHA PCA: 8/11 NCA: 0/59 | SHD PCA = 1/11 (CPC 1) NCA = 0/59 |
| **Tayal 2003**  n=20 | ROSC PCA = 9/12 NCA = 0/8 |  | SHD PCA = 7/12 NCA = 0/8 |
| **Salen 2001**  n=102 |  | SHA PCA first echo = 12/39 NCA first echo = 1/63  PCA any echo = 11/41  NCA all echos = 2/61  PCA all echos = 6/6  NCA any echo = 6/86 |  |
| **Blaivas 2001**  n=169 |  | SHA PCA = 20/33 NCA = 0/136 |  |

*Appendix 4.1. Forest plot of positive and negative likelihood ratio for TOR outcome in MCA group*

**
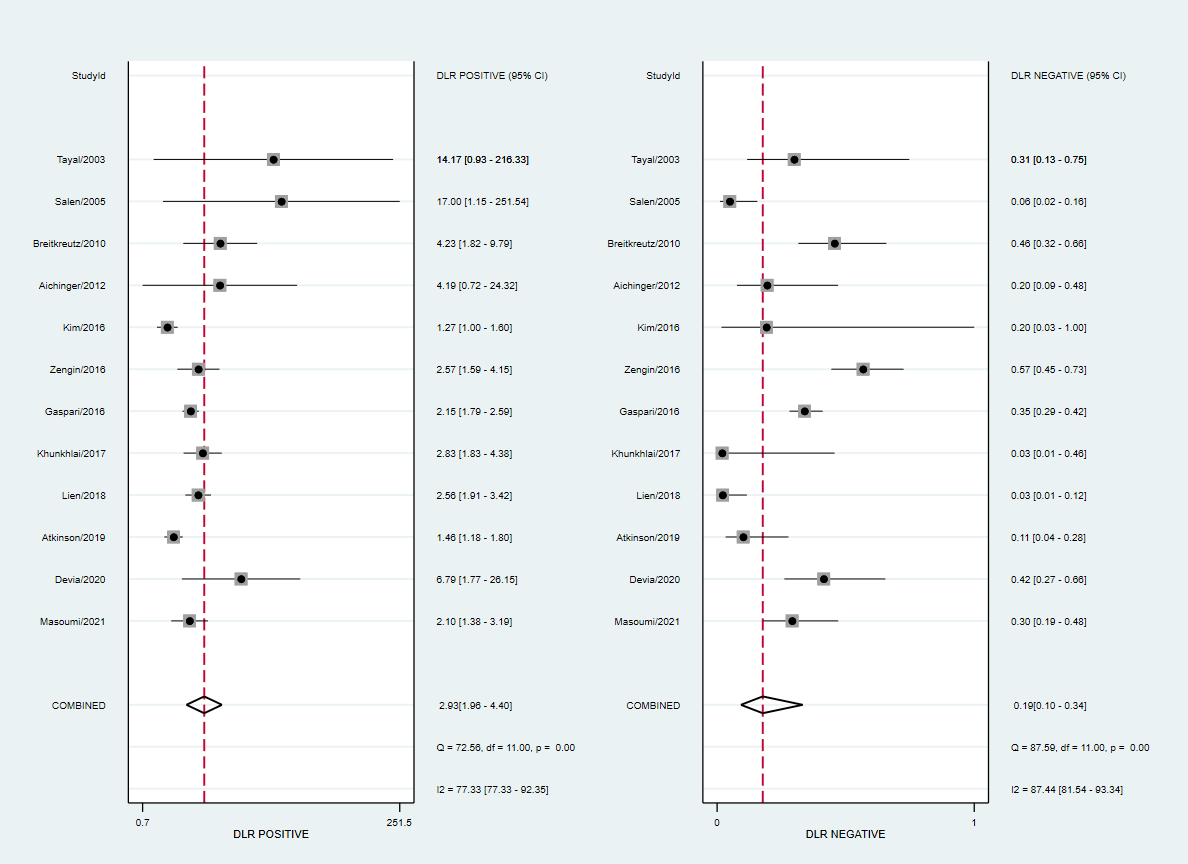
**

*Appendix 4.2. Forest plot of positive and negative likelihood ratio for STD outcome in MCA group*


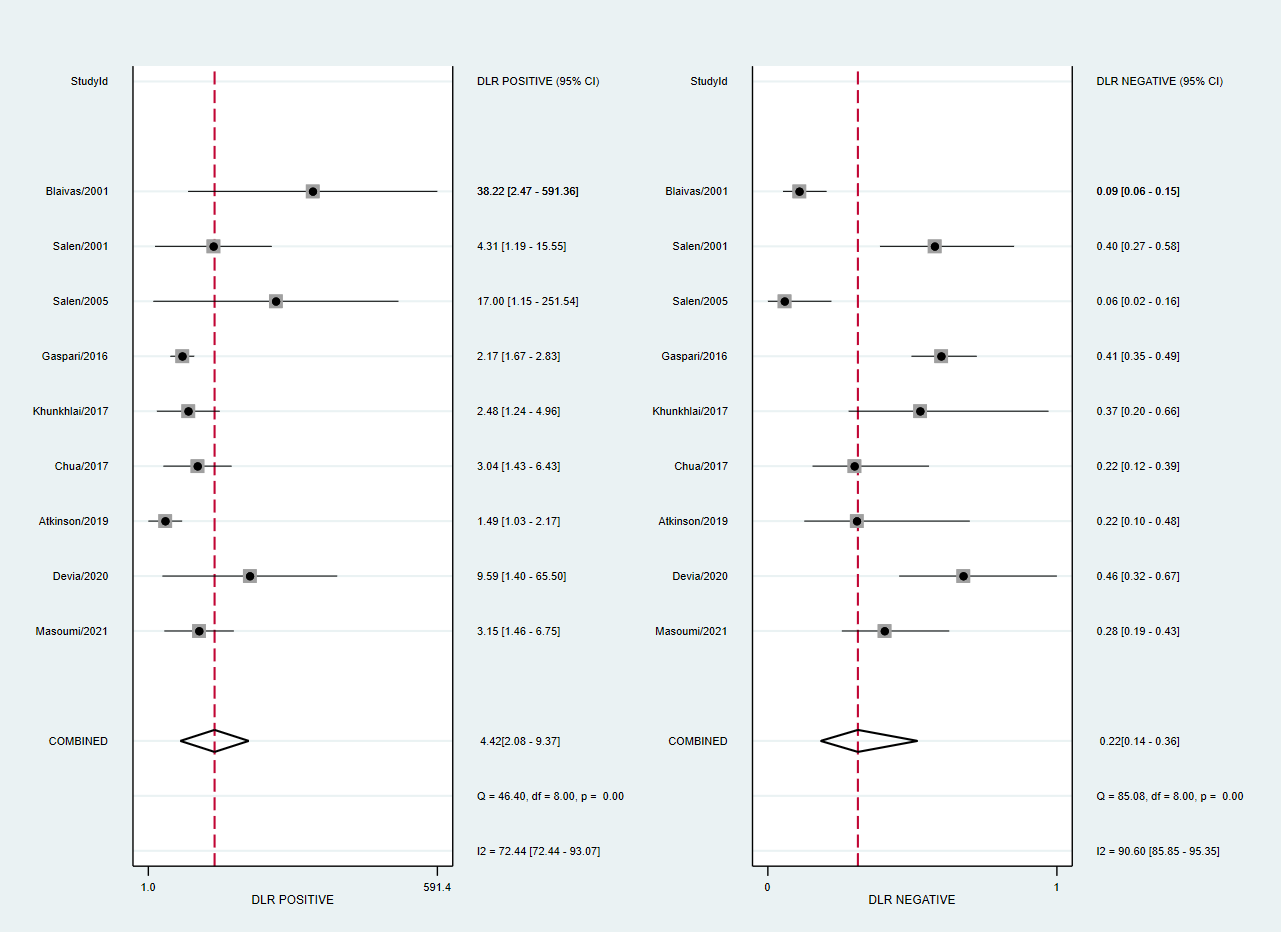


*Appendix 4.3. Forest plot of positive and negative likelihood ratio for LTD outcome in MCA group*


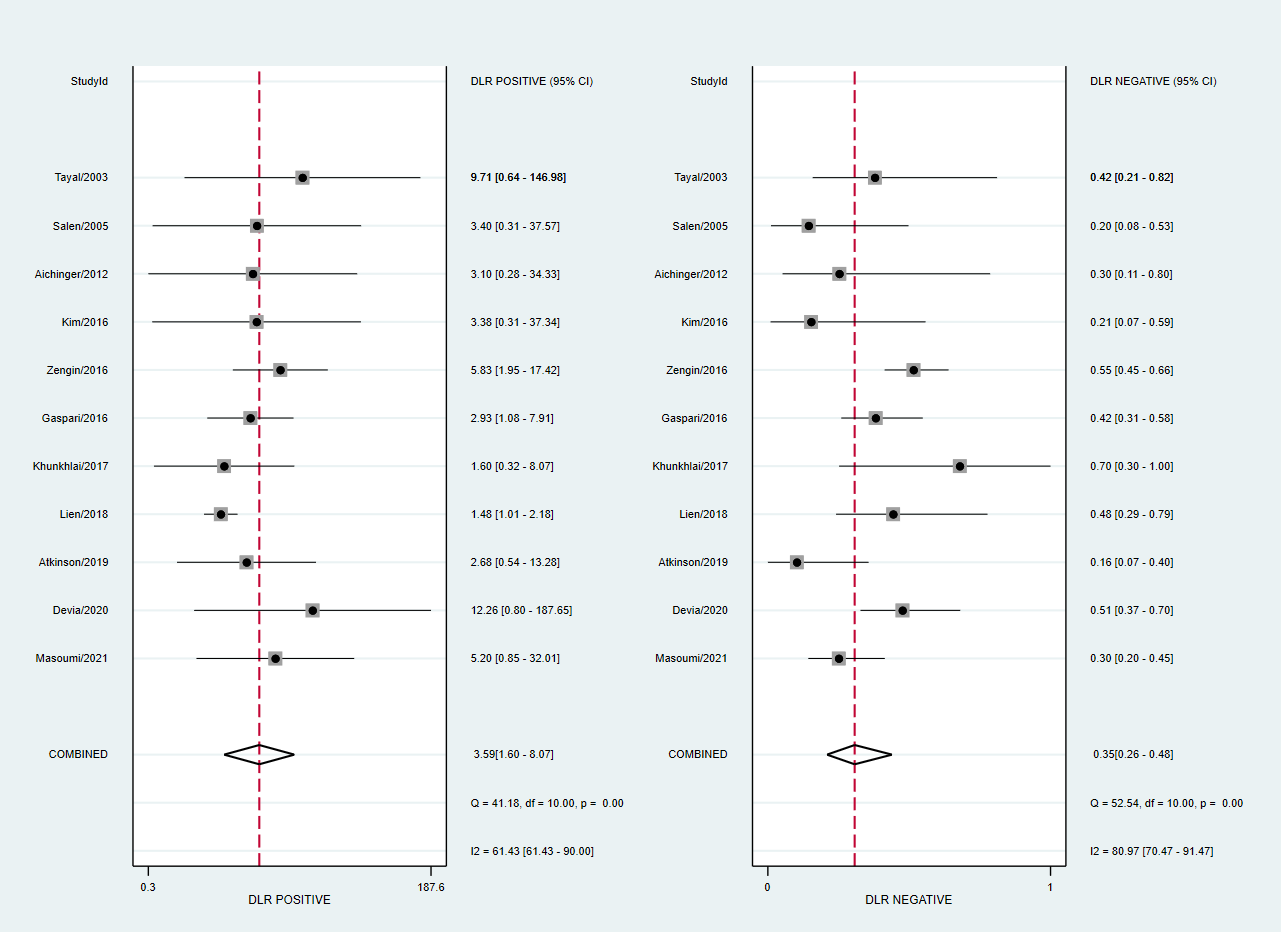


*Appendix 5.1. Deeks’ funnel plot for publication bias of TOR outcome in MCA group*


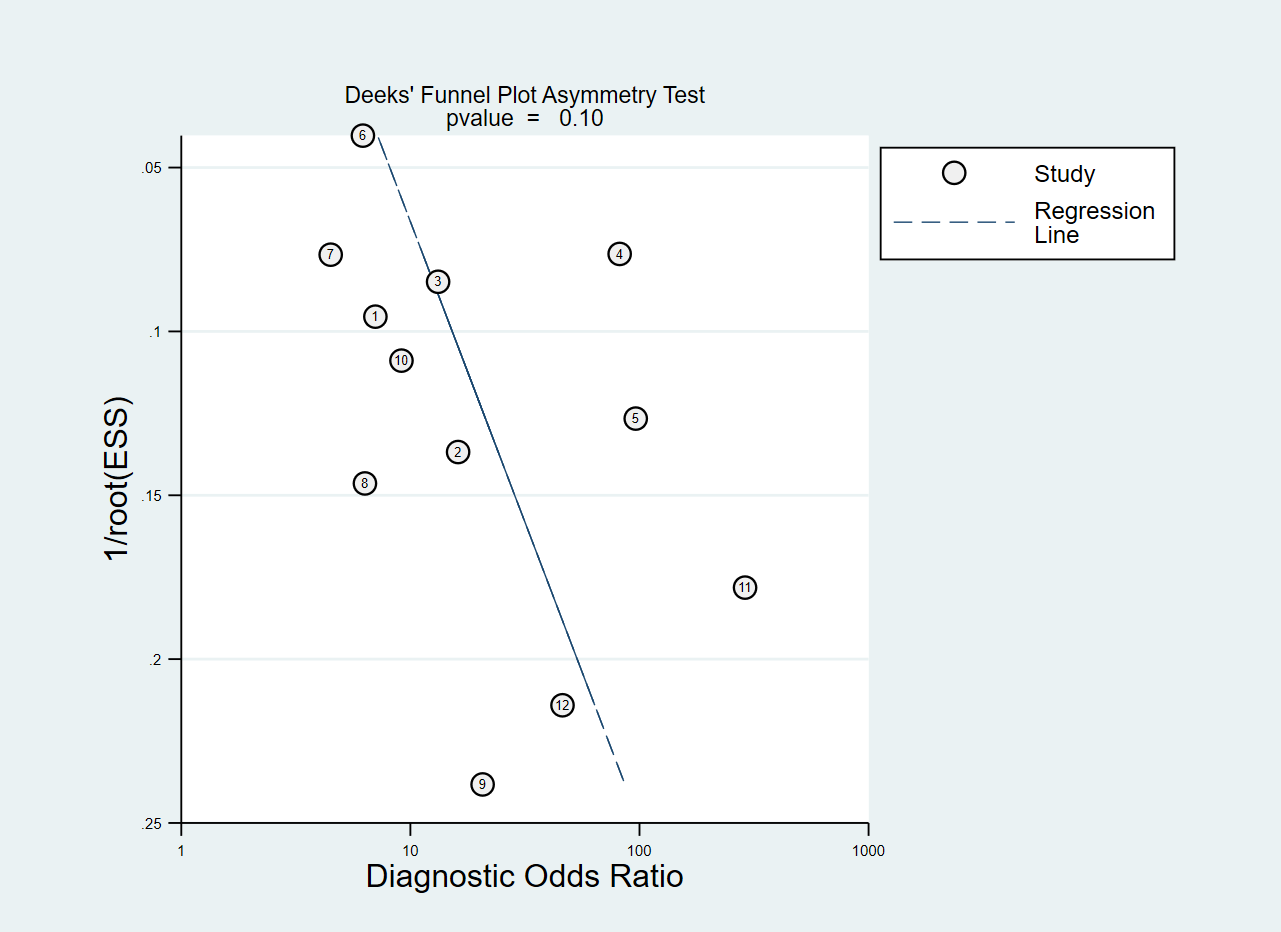


*Appendix 5.2. Deeks’ funnel plot for publication bias of STD outcome in MCA group*


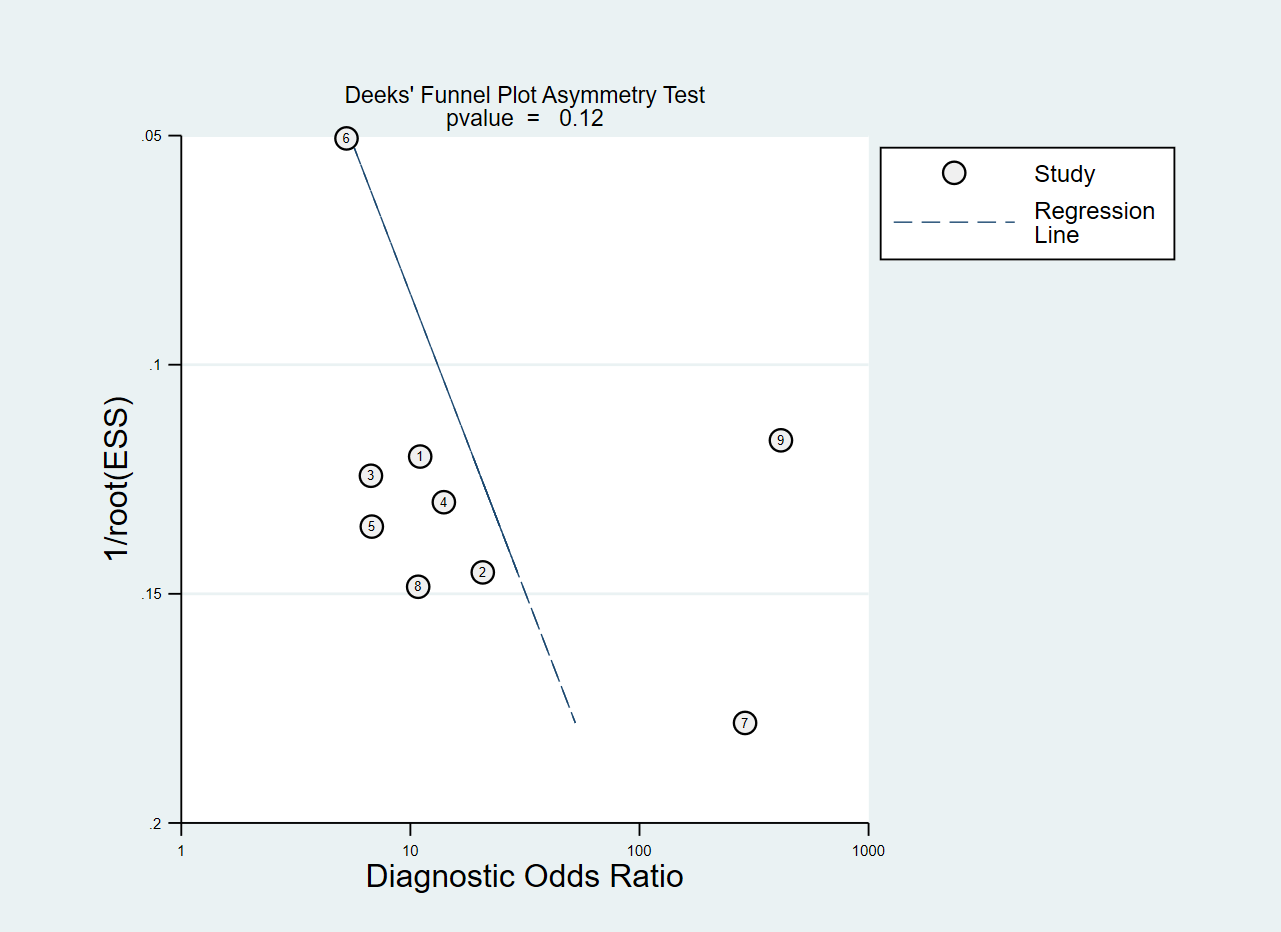


*Appendix 5.3. Deeks’ funnel plot for publication bias of LTD outcome in MCA group*


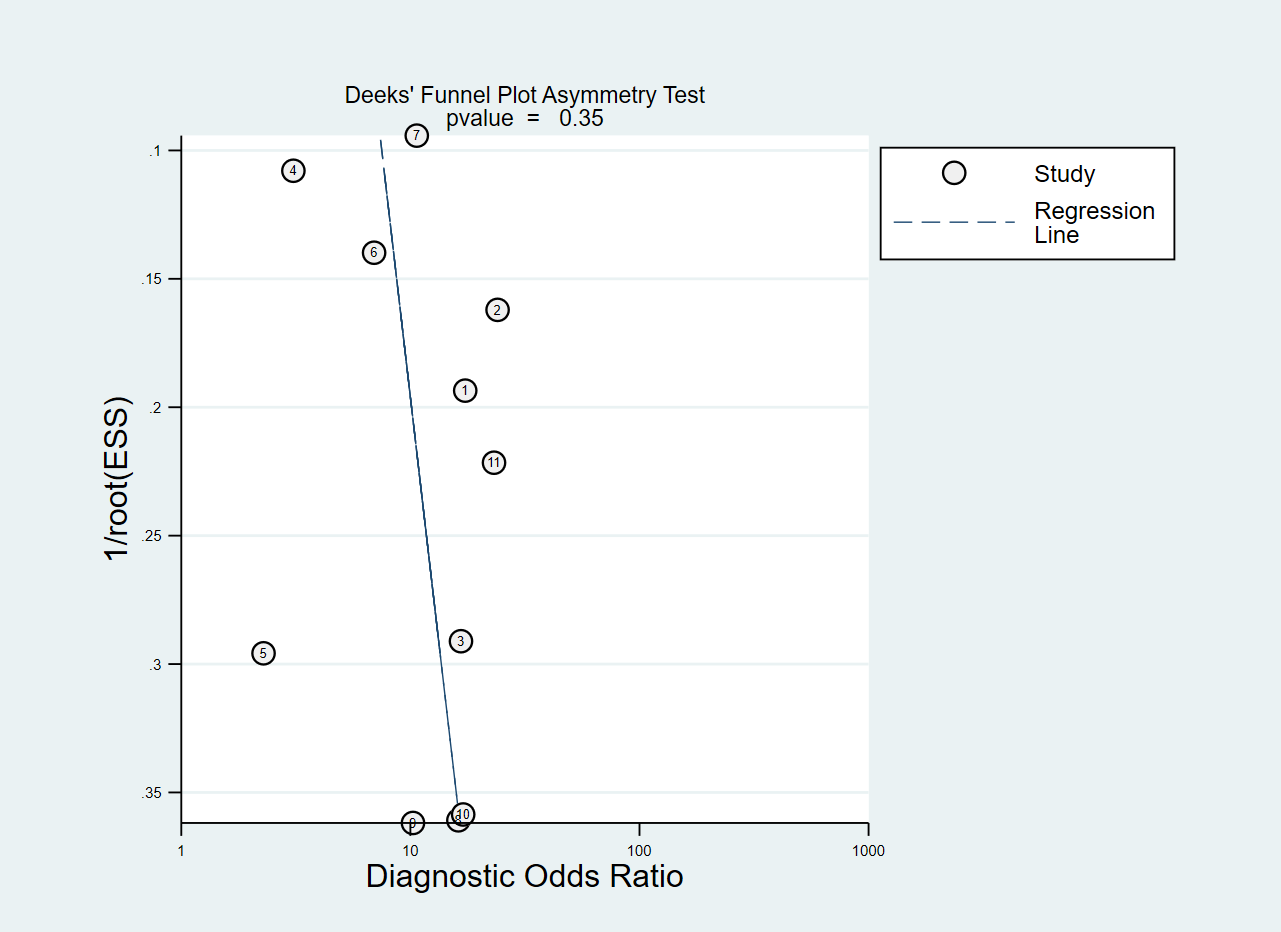

Supplement: Supplementary file 1 — Additional file 1. Appendix S1, S2, S3, S4, S5. [file 13089_2024_360_MOESM1_ESM.docx]
